# Early strong predictors of decline in instrumental activities of daily living in community-dwelling older Japanese people

**DOI:** 10.1371/journal.pone.0266614

**Published:** 2022-04-05

**Authors:** Yohei Masugi, Hisashi Kawai, Manami Ejiri, Hirohiko Hirano, Yoshinori Fujiwara, Tomoki Tanaka, Katsuya Iijima, Takashi Inomata, Shuichi P. Obuchi

**Affiliations:** 1 Tokyo Metropolitan Institute of Gerontology, Tokyo, Japan; 2 Institute of Gerontology, The University of Tokyo, Tokyo, Japan; Fondazione Santa Lucia Istituto di Ricovero e Cura a Carattere Scientifico, ITALY

## Abstract

**Objective:**

Our aim is to determine the strong predictors of the onset of instrumental activities of daily living (IADL) decline in community-dwelling older people.

**Design:**

A prospective cohort study with a two-year follow-up.

**Setting:**

Kashiwa City, Chiba Prefecture, Japan and Toshima Ward, Tokyo Metropolitan, Japan.

**Participants:**

The data were acquired from two cohorts. The final sample comprised 1,523 community-dwelling older people aged 65–94 years (681 men, 842 women). They were individuals who were independent in IADL at baseline and participated in follow-up IADL assessments two years later.

**Measurements:**

At baseline, comprehensive assessments were performed including: health interview, gait function, hand-grip strength, skeletal muscle mass, balance function, oral function, dietary lifestyle, cognitive function, quality of life, mental status, and social network. When the two-year follow-up was performed, IADL declines were observed in 53 out of 1,523 people. The association of each Z-transformed parameter with the occurrence of IADL decline was examined by employing a binominal logistic regression model adjusting for age, gender, body weight, body height, and medical history. An odds ratio (OR) and a 95% confidence interval were calculated and compared between different parameters.

**Results:**

A decrease in walking speed and one-legged stance time, whereas an increased timed up & go test time was associated with significant ORs for the occurrence of IADL decline.

**Conclusion:**

Gait-related parameters appear to be the strong predictors of the onset of IADL decline in community-dwelling older people.

## 1. Introduction

Maintaining sufficient mental and physical capacity is the key to successful aging. According to Lawton’s model, functional capacity can be divided into the following seven levels of competence: 1) life maintenance; 2) functional health; 3) perception-cognition; 4) physical self-maintenance; 5) instrumental self-maintenance; 6) effectance; and 7) social role [1]. Physical self-maintenance (i.e., basic activities of daily living; BADL) in particular represents basic functions that include performing routine activities of daily living such as eating, bathing, dressing, using the toilet and walking. Whereas, instrumental self-maintenance (i.e. instrumental activities of daily living; IADL) represents a more complex set of skills which require higher functioning compared to the BADLs. Examples of these include cooking, driving, using the telephone or computer, shopping, keeping track of finances, and managing medication. It was reported that BADL and IADL impairments were associated with negative outcomes such as days of hospitalization, the number of visits to the doctor, and the presence of dementia [2]. The 2-year occurrence of IADL impairments has been reported to be 5–20% in the older population in Japan [3]. Lawton’s model demonstrates that the presence of IADL impairments generally occurs before that of BADL impairments. Therefore, the prediction of IADL impairments appears to be more important than that of BADL impairments for early detection of functional decline in older people.

There are several indicators which may serve as predictors of the onset of IADL dependence in community-dwelling older people [4–13]. For example, a 6-year cohort study in a Japanese rural community showed that intellectual activities, self-rated health, chewing ability, and physical performance were identified as indicators for the prediction of IADL decline [12]. Another study reported that lower cognitive functioning was a significant predictor for IADL decline in community-dwelling non-disabled older people [9].Further, a cross-sectional study established that the current BADL disability may be caused by multiple factors such as health condition, body function, body structure, as well as personal factors [14]. Taking into account these results, there appears to be multiple factors that contribute to development of IADL impairment.

As described above, the predictors of the onset of IADL dependence in the older people has been well studied [4–13]. However, to our knowledge, no study exists that demonstrates which of these important indicators is the strong predictor of the onset of IADL decline. Determining this predictor may lead to the development of preventive practices. Therefore, the aim of this study is to investigate the association between each indicator (e.g., gait function, muscle strength, balance function, oral function, dietary lifestyle, cognitive function, quality of life, mental status, and social network) and the onset of IADL decline in order to determine the strong predictor for the onset of IADL decline in community-dwelling older people. The indicators investigated in this study included many indicators related to IADL impairment in previous studies [3–12]. In a systematic review, Vermeulen *et al*. proposed that in order to identify older people who need aid, a short-term predictive value of the indicators would be more substantial than a long-term predictive value [15]. Then, a previous study showed that the acceleration of cognitive decline occurs in a few years prior to the diagnosis of dementia [16]. Therefore, terminal IADL decline would also occur within a minimum of two years. Hence, the follow-up for this study took place two years after the initial assessment.

## 2. Methods

### 2.1. Ethics statement

The Toshima cohort study was approved by the ethics committee of Tokyo Metropolitan Institute of Gerontology (approval number 32 in 2014), while the Kashiwa study was approved by the ethics committee of the University of Tokyo (number 12–8). This ethics approval is in accordance with the Declaration of Helsinki (1964). All the participants in this study provided written informed consent.

### 2.2. Study population

**Fig 1:**
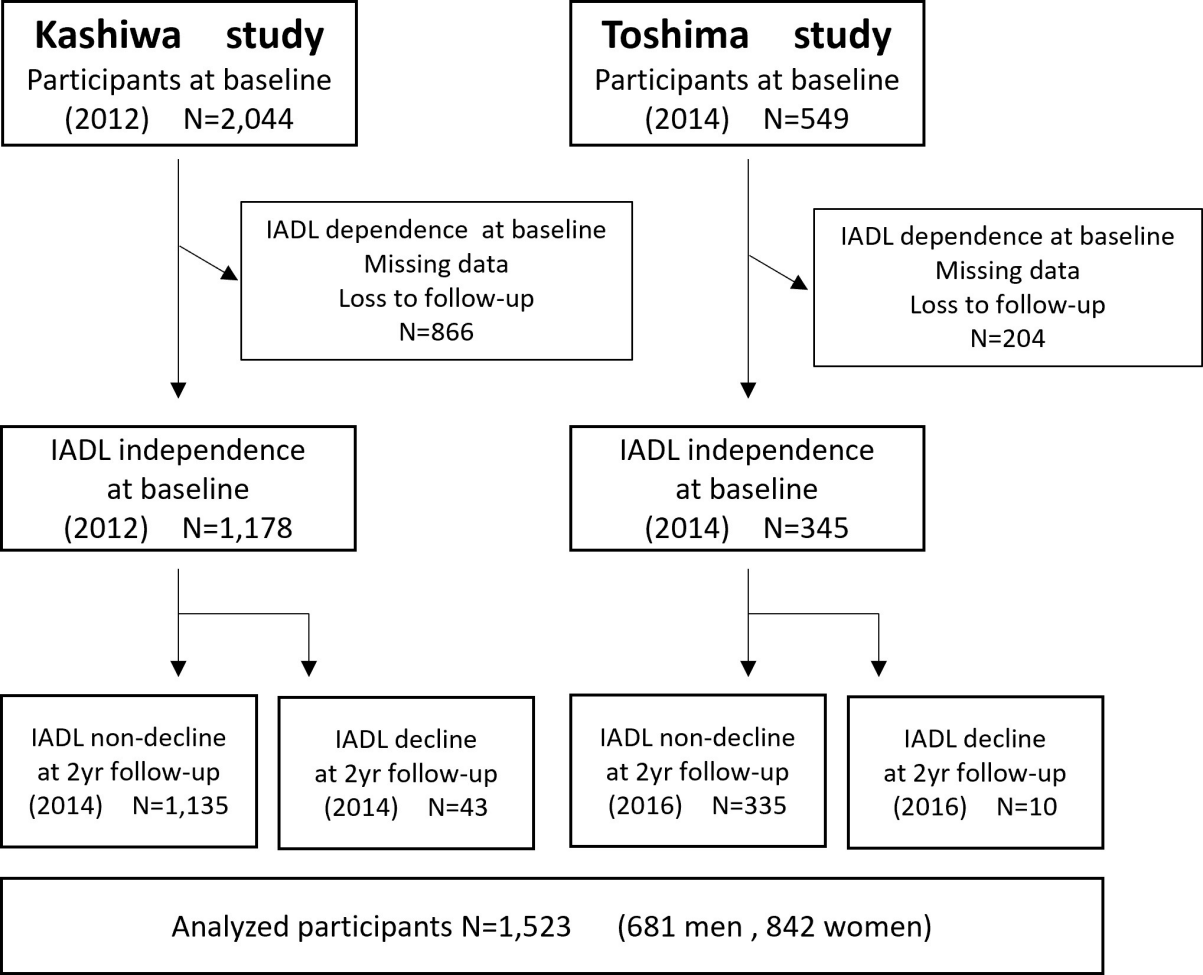
Flow diagram of participants analyzed in this study.

The data of two different cohorts in Japan was utilized. The first cohort was observed in the Kashiwa study which took place from 2012 to 2014. The second cohort was observed in the Toshima study from 2014 to 2016. Both studies were conducted to investigate age related functional changes including physical, cognitive and social functions in community-dwelling older population in Japan. The original papers have already reported studies on the Kashiwa [17] and Toshima cohorts [18]. The number of participants is shown in Fig 1. In the Kashiwa study, a total of 12,000 community-dwelling older people (aged 65 years or older) were randomly selected from the resident register of Kashiwa city, Chiba, Japan in 2012. They were notified through mail and 2,044 people agreed to participate in the Kashiwa study. In the Toshima study, a total of 6,158 community-dwelling older people (between the ages of 65 to 84 years) were selected. These participants lived in a part of the Toshima Ward and were not nursing home residents. Each participant was notified via mail about participating in the surveys, including a health interview, body composition, movement function, oral function, and mental function. A total of 549 individuals participated in the Toshima study. In these two cohorts, 1,070 participants were excluded from the analysis because of missing data related to IADL, loss to follow-up, and IADL dependence at baseline. The analyzed sample was composed of 1,523 community-dwelling older people. They were individuals who were independent in IADL at baseline survey and participated in follow-up IADL assessments two years later.

### 2.3. IADL status

The Tokyo Metropolitan Institute of Gerontology Index of Competence (TMIG-IC) was employed to evaluate the IADL status in participants. TMIG-IC was developed to assess the following three high-level of functional capacities of older people in Japan: (1) IADL, (2) Intellectual activity, and (3) Social role. The IADL list of TMIG-IC consisted of five questions (Table 1). The participants were asked to respond with either a “yes” (1 point) or “no” (0 points). An IADL score between 0–5 was calculated based upon the total points of the five questions. In this study, IADL dependence was defined as ≦4 IADL scores based on a previous study using TMIG-IC [12].

**Table 1:** Instrumental activities of daily living (IADL) subscale of The Tokyo Metropolitan Institute of Gerontology Index of Competence (TMIG-IC).

Questionnaires	Yes	No
1. Can you use public transportation (bus or train) by yourself?	1	0
2. Are you able to shop for daily necessities?	1	0
3. Are you able to prepare meals by yourself?	1	0
4. Are you able to pay bills?	1	0
5. Can you handle your own banking	1	0

The present study used the questionnaires translated from English into Japanese.

The total score (0–5) is calculated as the sum of the five items.

The medical history of the participants in regard to chronic diseases such as hypertension, stroke, diabetes mellitus, hyperlipemia, chronic renal failure, heart disease, and cancer was recorded by nurses.

### 2.4. Physical structure and function

Body height (cm), body weight (kg), body mass index (BMI) (kg/m^2^), and appendicular skeletal mass index (ASMI) (kg/m^2^) were measured as physical structure measurements. ASMI (kg/m^2^) was estimated by using a bioelectrical impedance analysis system (Kashiwa study: InBody 430, InBody, Seoul, Korea; Toshima study: InBody 720, InBody, Seoul, Korea).

Physical function was measured through the following assessments: hand-grip strength (kg), one-legged stance time (s), usual walking speed (m/s), maximum walking speed (m/s), and timed up & go (TUG) time (s) [19,20]. A description of the cutoff scores can be found in a previous study [19]. Hand-grip strength was measured as an index of muscle strength. A hand dynamometer (Kashiwa study: Takei Scientific Instruments, Niigata, Japan; Toshima study: AS ONE Corporation, Osaka, Japan) was used for measurements. Grip strength was measured twice, and a higher value was used for the analysis. One-legged stance time was measured with the participant’s eyes open. The maximum measurement time was 60 s. The measurement was terminated when the time exceeded 60 s. Participants carried out two trials, and the better time was used. Walking time was measured over the central 5 m of an 11 m straight course. Speed was calculated from walking time and distance. Usual walking speed was measured once. Maximum walking speed was measured twice, and the better of the two results was used. TUG time was measured using a chair and cone placed at a distance of 3 m from the chair. The participants were instructed to move around the cone as quickly as possible and return to the chair. The participants were asked to start walking when they were ready.

### 2.5. Oral and nutritional function

Oral function was assessed by a speech diadochokinetic test (i.e., alternating motion rate; AMR) [18]. For the test, participants had to perform a rapid repetition of a single syllable (/ta/) for 10 seconds. The count was measured by a device called the KENKOU-KUN handy (Takei Scientific Instruments, Niigata, Japan). The count per second was calculated following the measurement. Swallowing function was assessed by the repetitive saliva swallowing test (RSST). For this test, participants were instructed to swallow their own saliva repeatedly for a maximum of 30-seconds, as quickly as they could. The experimenter measured the time (s) taken for the first swallow as RSST1.

Dietary habits were evaluated by using the Dietary Variety Score (DVS). This index was developed by Kumagai *et al*. (2003) and has been used to assess the dietary habits of older people in Japan [21]. The participants were asked to answer a 10-item self-report questionnaire related to their food intake. The total number of food groups consumed daily was calculated. The total score for the DVS ranged from 0 to 10. A higher score indicates a higher level of dietary variety.

### 2.6. Mental and cognitive function

Depressive symptomatology was evaluated using the Japanese version of the Geriatric Depression Scale (GDS) [22]. This assessment included a 15-item self-report questionnaire to identify symptoms of depression in older people. The participants were asked to answer with a simple “yes” or “no.” The total score of the GDS ranged from 0 to 15. A higher GDS score indicates a higher level of depressive symptoms.

The WHO-Five Well-being Index (WHO-5) was used to measure the current subjective Quality of Life (QOL) which assesses positive mood, vitality, and general interest [23]. The test includes a 5-item self-report questionnaire, which the participants are asked to answer in six stages (0 to 5 points). The total score for the WHO-5 ranged from 0 to 25. A higher score indicates a higher level of QOL.

A Mini-Mental State Examination (MMSE) was performed to evaluate the cognitive functioning among the older people [24]. The score for the MMSE ranged from 0 to 30. A higher score indicates a higher level of cognitive function.

### 2.7. Social networks

The Lubben Social Network Scale (LSNS-6) was used to measure social engagement including that of family and friends [25]. This test includes a 6-item self-report questionnaire in which the participants were asked to answer in six stages (0 to 5 points). The score for LSNS-6 score ranged from 0 to 30. A higher score indicates a higher level of social engagement.

### 2.8. Statistical analysis

A Pearson chi-square test and a Mann-Whitney U test were employed to compare baseline characteristics between the IADL decline and IADL non-decline groups. The Mann-Whitney U-test was performed to compare the baseline parameters between the dropout (n = 796) and non-dropout (n = 1523) groups. Neither those who dropped out nor those who did not have any impairment in IADL at baseline. Binomial logistic regression analyses (by the forced entry method) were performed using either “with IADL decline” or “without IADL decline” as the dependent variable. Each parameter served as the predictor variable. These analyses were then adjusted according to baseline age, gender, body weight, body height, and medical history of illnesses (e.g., hypertension, stroke, diabetes mellitus, hyperlipemia, chronic renal failure, heart disease, and cancer). The statistical results were shown as an odds ratio (OR) with a 95% confidence interval (CI). To compare the results of each predictor variable, a Z-transformation was applied to each parameter prior to the binominal logistic regression analysis. A Hosmer-Lemeshow test was used to assess the fit of the logistic regression model with *P* < 0.05 as the significance level. All statistical analyses were performed using the SPSS software (IBM, Chicago, USA).

## 3. Results

Table 2 shows the baseline characteristics of the study participants using the median and interquartile range (IQR). After a two-year follow-up period, the functional decline of IADL was observed in 53 out of 1,523 participants. A Mann-Whitney U test revealed significant differences in the variables (i.e., age, one-legged stance time, usual walking speed, maximum walking speed, TUG time, AMR, MMSE, and LSNS-6) between the participants of IADL decline and IADL non-decline groups (*P* < 0.05). Additionally, the Pearson chi-square test showed a significant difference in percentage of gender and percentage of history of cancer between the participants of IADL decline and IADL non-decline groups (*P* < 0.05).

**Table 2:** Baseline characteristics of participants of IADL decline and IADL non-decline groups.

Variables		IADL decline (n = 53)	IADL non-decline (n = 1470)	*P*-value
Cohort				
-Kashiwa	N	43	1135	
-Toshima	N	10	335	
Age (years)	median [IQR]	75 [70 to 78]	72 [68 to 76]	*P* = 0.018
Gender				
-men	% (N./Total)	66.0 (35/53)	43.9 (646/1470)	*P* = 0.001
Body height (cm)	median [IQR]	160.2 [153.4 to 165.8]	156.5 [150.5 to 163.5]	*P* = 0.064
Body weight (kg)	median [IQR]	56.8 [49.6 to 65.8]	55.6 [49.1 to 63.0]	*P* = 0.415
Body Mass Index (kg/m^2^)	median [IQR]	22.1 [20.9 to 24.1]	22.6 [20.7 to 24.6]	*P* = 0.585
History of				
hypertension	Present, % (N./Total)	49.1 (26/53)	42.6 (626/1470)	*P* = 0.350
stroke	Present, % (N./Total)	3.8 (2/53)	6.9 (101/1469)	*P* = 0.377
diabetes mellitus	Present, % (N./Total)	11.3 (6/53)	11.6 (171/1470)	*P* = 0.945
hyperlipemia	Present, % (N./Total)	39.6 (21/53)	42.0 (618/1470)	*P* = 0.350
chronic renal failure	Present, % (N./Total)	1.9 (1/53)	0.5 (8/1467)	*P* = 0.211
heart disease	Present, % (N./Total)	17.0 (9/53)	17.4 (256/1470)	*P* = 0.935
cancer	Present, % (N./Total)	24.5 (13/53)	14.4 (211/1470)	*P* = 0.04
ASMI (kg/m^2^)	median [IQR]	6.7 [5.7 to 7.3]	6.4 [5.7 to 7.3]	*P* = 0.574
Hand-grip strength (kg)	median [IQR]	28.5 [23.0 to 34.8]	26.0 [22.0 to 34.0]	*P* = 0.356
One-legged stance time (s)	median [IQR]	30.5 [11.3 to 60.0]	60.0 [29.8 to 60.0]	*P* < 0.001
Usual walking speed (m/s)	median [IQR]	1.4 [1.2 to 1.6]	1.5 [1.3 to 1.6]	*P* = 0.003
Maximum walking speed (m/s)	median [IQR]	2.0 [1.8 to 2.2]	2.1 [1.9 to 2.4]	*P* = 0.009
TUG time (s)	median [IQR]	5.8 [5.1 to 7.2]	5.4 [4.8 to 6.0]	*P* = 0.001
AMR (/s)	median [IQR]	6.0 [5.1 to 6.4]	6.2 [5.6 to 6.8]	*P* = 0.018
RSST1 (s)	median [IQR]	2.0 [1.0 to 3.0]	2.0 [1.0 to 3.0]	*P* = 0.77
DVS (score) (Score range from 0 to 10)	median [IQR]	4.0 [2.0 to 6.0]	4.0 [3.0 to 5.0]	*P* = 0.704
GDS (score) (Score range from 0 to 15)	median [IQR]	1.5 [0 to 4.0]	2.0 [0.0 to 4.0]	*P* = 0.719
WHO-5 (score) (Score range from 0 to 25)	median [IQR]	18.0 [15.0 to 22.0]	19.0 [16.0 to 21.0]	*P* = 0.367
MMSE (score) (Score range from 0 to 30)	median [IQR]	28.0 [27.0 to 29.0]	29.0 [28.0 to 30.0]	*P* = 0.003
LSNS-6 (score) (Score range from 0 to 30)	median [IQR]	14.0 [10.0 to 19.0]	16.0 [13.0 to 20.0]	*P* = 0.038

ASMI: Appendicular skeletal mass index. TUG: Timed up & go. AMR: Alternating motion rate.

RSST: Repetitive saliva swallowing test. DVS: Dietary Variety Score. GDS: Geriatric Depression Scale.

WHO-5: WHO-Five Well-being Index. MMSE: Mini-Mental State Examination.

LSNS-6: Lubben Social Network Scale. IQR: Interquartile range.

**Fig 2:**
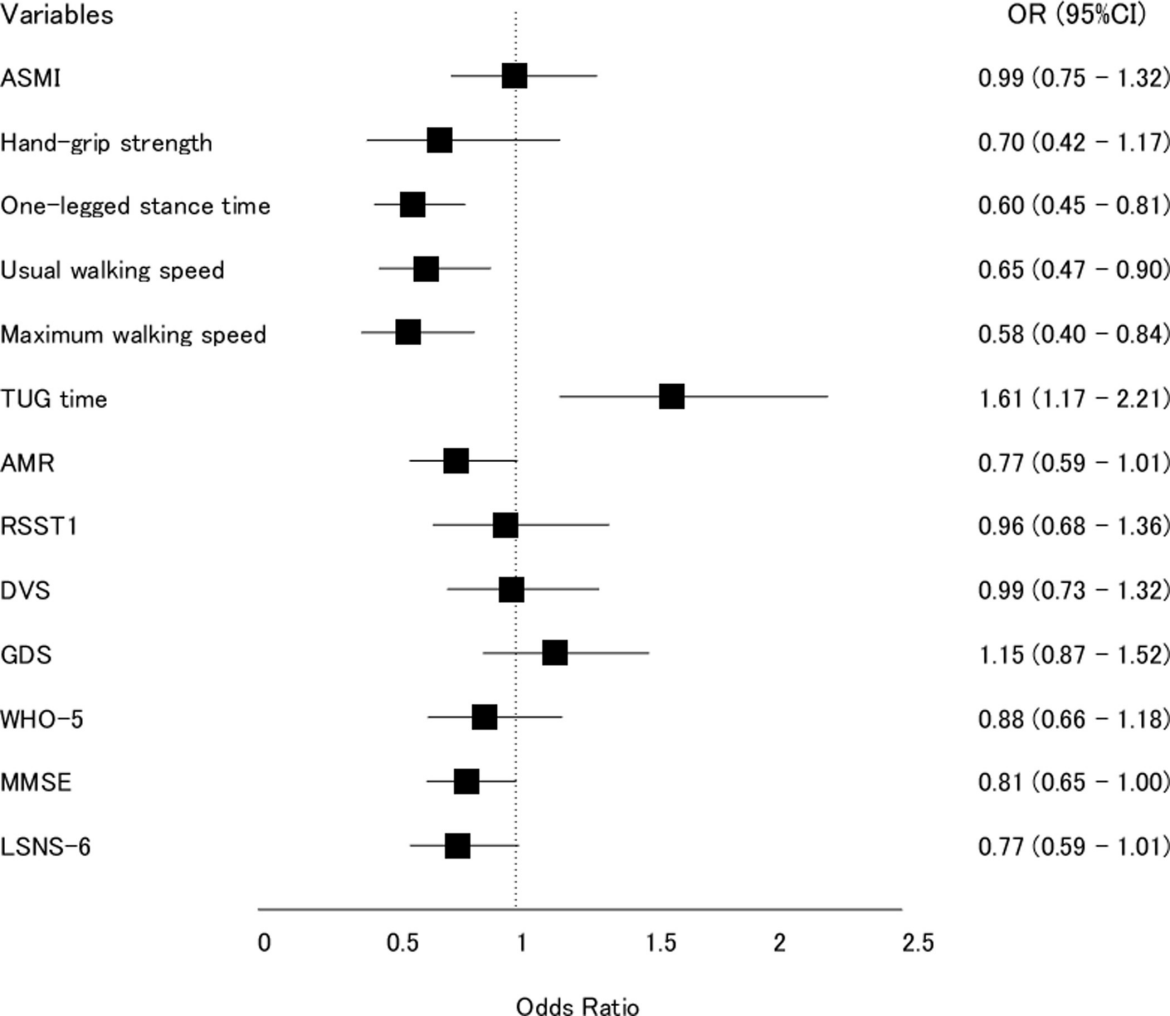
Odds ratios (ORs) and 95% confidence intervals for the association of each variable at the baseline survey with IADL decline after two years. All the ORs were adjusted for baseline age, gender, weight, height, and medical history. Abbreviations: ASMI, appendicular skeletal mass index; TUG = timed up & go; AMR = alternating motion rate; RSST = repetitive saliva swallowing test; DVS = dietary variety score; GDS = Geriatric Depression Scale, WHO-5 = World Health Organization-Five Well-being Index; MMSE = Mini-Mental State Examination; LSNS-6 = Lubben Social Network Scale.

Fig 2 shows the ORs and 95% CIs of each Z-transformed parameter. The Hosmer-Lemeshow test showed that all the logistic regression models were fitted (*P* > 0.05). Four of the variables (i.e., one-legged stance time, usual walking speed, maximum walking speed, and TUG time) were significantly associated with the occurrence of IADL decline (*P* < 0.05), and showed larger ORs than the other variables.

The Mann-Whitney U-test was performed to compare the baseline parameters between the dropout (n = 796) and non-dropout (n = 1523) groups. There was no significant difference in age between the dropouts and non-dropouts. The dropout group had lower motor function than the non-dropout group (hand-grip strength, *P* = 0.028; one-legged stance time, *P* < 0.001; maximum walking speed, *P* < 0.001; TUG time, *P* < 0.001).

## 4. Discussion

The present study revealed that gait-related parameters such as maximum walking speed, usual walking speed, and TUG time were the early strong predictors of the occurrence of IADL decline in community-dwelling older people. In addition, another parameter, the one-legged stance time related to the balance functioning, was also an indicator associated with the occurrence of IADL decline. These results are discussed in detail below.

### 4.1. Methodological considerations

A binominal logistic regression analysis was used, calculating ORs and 95% Cls to determine which predictors are significant in determining the onset of IADL decline in community-dwelling older people. These analyses were performed after adjusting for potential confounders like age, gender, body weight, body height, and medical history. Ensuing this, the Hosmer-Lemeshow tests showed good fit for all logistic regression models. Therefore, based on these results, each logistic regression model used in this study appeared to be valid.

Each parameter was standardized with a Z-score prior to conducting the logistic regression analyses allowing for the comparison of each parameter which previously had different units. We believe that these statistical procedures could reveal the possibility of a significant association between various predictor variables and the onset of IADL decline in community-dwelling older people.

### 4.2. Predictors of future IADL decline

In the present study, the 2-year occurrence of IADL impairment was 3.5%, which was low compared with reports from other countries [26]. In a previous study conducted 20 years ago in Japan, the 2-year occurrence of IADL impairments was 5–20% [3]. Furthermore, the IADL scores of older people in Japan have been improving [27]. Based on these reports, the 2-year occurrence of IADL impairments in this study is considered a reasonable result in Japan.

Previous studies have shown that many variables are associated with physical function, cognitive function, and social role. These are often identified as the predictors of future IADL decline in the older people [8,9,12,13]. These predictors are important for the development of preventive aid and practices. However, the indicators that are the most significant predictors of the future IADL decline are unknown. By standardizing the variables, the ORs were compared among each variable in this study. The current results showed that indicators of gait-related parameters (i.e., maximum walking speed, usual walking speed, and TUG time) are strongly associated with the occurrence of IADL decline in the older people. The TUG time, in particular, was a more significant predictor compared to the maximum and usual walking speed. The TUG test included standing, walking, turning and sitting. These movements require higher balance function. Although maximum and usual walking speed are significant predictors, other elements such as standing, turning, and sitting are also considered important factors in predicting future IADL decline. In this study, IADL status was examined by using TMIG-IC which was established by Koyano *et al*. (1991) in Japan. The TMIG-IC related to IADL status includes more complex daily activities such as transportation, shopping, meal preparation and money management compared to the more basic daily activities [28]. The ability to go out of the house is crucial in achieving these activities and it is found that the indicators of gait-related parameters were more strongly associated compared to the other parameters analyzed in this study. The current findings indicate that walking as a function is the most essential assessment in detecting future IADL decline in older people, supporting the statement that gait speed is a vital sign in prediction [29]. Maintaining gait functionality might lead to preventing future IADL decline.

This study found that parameters related to gait were important for predicting the occurrence of IADL impairments. Although we did not measure gait patterns, biomechanical and neurophysiological studies have shown age-related changes in gait patterns [30–32]. Therefore, it is likely that similar changes occurred in older people with a decline in IADL in this study.

Several studies reported that a decrease in cognitive function is related to the decline of BADL and IADL [6,11]. Cognitive function assessed by MMSE can be a predictor of the onset of BADL and IADL decline in community-dwelling older people [8]. However, there is no study showing its effectiveness as a short-term predictor. As a result, the present study showed that the MMSE score is not statistically nor significantly associated with IADL decline. Since the intact cognitive functioning slowly declines for about 10 years until the development of mild cognitive impairment [33], it is likely that a decline in cognitive function could not detect immediate future IADL decline.

The previous study shows that the social role performance assessed by TMIC-IG is significantly associated with future IADL decline [8]. Lawton’s hierarchical model, which has been accepted as a theoretical framework, comprises the seven sublevels of competence: 1) life maintenance; 2) functional health; 3) perception and cognition; 4) physical self-maintenance; 5) instrumental self-maintenance; 6) effectance; and 7) social role. Based on these models, the competence of higher order should decline first and progressively get worse with age. Social role performance should be impaired prior to IADL decline and should therefore be a predictor of future IADL decline in older people. However, the present results showed that social isolation (evaluated using LSNS-6) was not significantly associated with future IADL decline. Since social role performance required a much higher competence than instrumental self-maintenance [28], it is likely that the social role performance could not detect future IADL decline.

Of late, oral functioning has attracted attention from researchers and practitioners. Previous studies show that poor oral health is significantly associated with physical frailty, sarcopenia, and mortality among the community-dwelling older people [17]. However, the present results show that the tongue motor control (evaluated using AMR-ta) was not highly associated with future IADL decline. Oral function is not directly associated with the skills needed for instrumental self-maintenance. Therefore, it is likely that oral function could not detect the future IADL decline.

### 4.3. Limitations

This study had several limitations. 1) The present data were obtained from Japanese suburban areas (i.e., Kashiwa and Toshima); thus, the present results may differ in rural areas as well as other countries. 2) The proportion of participants with IADL decline was small; hence, the participants with IADL decline may have dropped out of the follow-up assessment. The motor function of the dropouts was lower than those who did not drop out. Although a power analysis was performed to confirm that the number of samples was large enough, generalization of the results needs careful consideration. Indicators of relatively low significance (i.e., cognitive function, social function, and oral function) may become significant when more representative samples are included. Thus, cognitive, social, and oral functions could be used as secondary predictors of future IADL decline in the older people. 3) A comprehensive assessment was performed in this study, which concluded that gait-related parameters were the strongest predictors of future IADL decline. However, the unexamined measures, such as socio-demographic factors, may provide results different from those of the present study. 4) The final sample size was very small compared with the total number of participants recruited. Although the participants were randomly selected, sample selection bias may have occurred in this study.

## 5. Conclusion

In conclusion, this study revealed that gait-related parameters such as TUG time, maximum walking speed, and usual walking speed are significantly associated with future IADL decline in community-dwelling older people. Daily monitoring of these indicators associated with gait-function would be useful for detecting future risk of IADL decline.
